# Whole-genome sequencing identifies potential candidate genes for egg production traits in laying ducks (*Anas platyrhynchos*)

**DOI:** 10.1038/s41598-022-21237-w

**Published:** 2023-02-01

**Authors:** Yanfa Sun, Yeqiu Zhang, Qiong Wu, Rulong Lin, Hongping Chen, Min Zhang, Jiaqi Lin, Enrong Xu, Meng Li, Yicheng Cai, Fan Deng, Wen Yue, Haozhe Pan, Xiaobing Jiang, Yan Li

**Affiliations:** 1 College of Life Sciences, Longyan University, Xinluo District, No. 1, Dongxiao North Road, Longyan, 3640012 Fujian People’s Republic of China; 2 Fujian Provincial Key Laboratory for the Prevention and Control of Animal Infectious Diseases and Biotechnology, Longyan, 3640012 Fujian People’s Republic of China; 3 Key Laboratory of Preventive Veterinary Medicine and Biotechnology, Longyan University, Longyan, 3640012 Fujian People’s Republic of China; 4 Longyan Shan-Ma Duck Original Breeding Farm, Agricultural Bureau of Xinluo District, Longyan, 364031 Fujian People’s Republic of China; 5 Fujian Provincial Animal Husbandry Headquarters, Gulou District, No. 24, Yeshan Road, Fuzhou, 3640012 Fujian People’s Republic of China

**Keywords:** Next-generation sequencing, Genetics, Animal breeding

## Abstract

Egg production traits are economically important in laying ducks. Genetic molecular mechanisms and candidate genes underlying these traits remain unclear. In this study, whole genome variants were identified through whole-genome resequencing using three high-egg producing (HEN) and three low-egg producing (LEN) laying ducks. The gene ontology (GO) terms and Kyoto Encyclopedia of Genes and Genome (KEGG) pathways for the genes of common differential variants between HEN and LEN ducks were determined. Frizzled class receptor 6 (*FZD6*) was further genotyped using the Sequenom MassARRAY iPLEX platform. The association of *FZD6* gene polymorphisms with 73 egg production and weight traits in 329 female ducks were estimated. A total of 65,535 single nucleotide polymorphisms (SNPs) and 4,702 indels were identified across the genome. Fourteen GO terms and 14 KEGG pathways were determined for the genes of common differential variants, including MAPK signaling, Wnt signaling, melanogenesis and calcium signaling pathways, which are key functional pathways for poultry egg production reported in previous reports. Further analysis showed that 27 SNPs of *FZD6* were associated with three early egg production of duck and egg weight traits, including egg production at 17 weeks (EP17), 18 weeks (EP18) and 19 weeks (EP19) and egg weight at 59 weeks (EW59). The *FZD6* should be considered a novel candidate gene for egg production traits in laying ducks.

## Introduction

In the modern poultry industry, egg production traits, including egg number (EN), egg weight (EW), and age at first egg (AFE), are important reproductive and economic factors^1,2^ and improved egg production traits are usually the goals of poultry breeding companies^3^. In chickens, egg production traits are quantitative traits whose genetic architecture has been uncovered using a quantitative trait locus (QTL) analysis in a previous genome-wide association study (GWAS)^4^. Over 659 QTLs for egg-production traits have been found^5^ and many promising genes and mutants for egg-production traits have been identified^6^. In contrast the genetic basis underlying the egg production traits in ducks is still not fully understood, because the heritability of egg production traits is low to intermediate, ranging from 0.13 to 0.43^7^. Some candidate genes for egg production traits have been investigated, mainly focused on the genes associated with reproductive physiology, such as the follicle-stimulating hormone receptor gene^8^, melatonin receptor gene^9^ and the prolactin gene^10^. However, candidate gene method has limited ability to identify genetic basis of egg production traits in duck.

With the development of proteomic technologies, it is possible to investigate the genetic basis for key traits of livestock and poultry by whole-genome resequencing. In pigs, 13,955,609 SNPs and 2,666,366 indels have been found and the common differential SNPs and indels between five high- and five low-prolificacy Yorkshire sows using whole-genome resequencing technology and several differential variants within candidate genes for reproduction traits were confirmed by comparing selective regions and published quantitative trait locus (QTL) data^11^. In chickens, whole-genome resequencing from eight chickens with slow- and fast-feathering rates showed 54,984 SNPs and mutated genes were involved in the response to stimuli, growth and reproduction processes and two SNPs involved in feather development were identified in the exonic regions of the Wnt signaling gene^12^. In ducks, selection signatures were uncovered by genome-wide comparisons among Mallards, indigenous-breeds and Pekin ducks and two selective sweeps for two important economic traits of the Pekin duck were identified through further fine-mapping based on resequencing of more than 1,000 ducks from an F2 segregating population generated by wild crossed with domestic birds^13^. In geese, the *KIT* gene underlying white or gray plumage color in Chinese domestic geese was identified by resequencing the whole genome of 18 geese from six populations, including white and gray goose breeds^14^.

There are 250–300 million Shan-ma ducks (*Anas Platyrhynchos*) raised every year and as one of the main Chinese indigenous egg laying duck types, makes up about half laying duck population raised in China. The breed originated from Longmen town, in Longyan city and in January 2017, the Ministry of Agriculture and Rural Affairs of China officially approved the registration and protection of agricultural products based on the Longyan Shan-ma duck from this region.

In this study whole-genome resequencing was performed on three full-sib high- and three full-sib low-egg producing Shan-ma ducks. The study aimed to identify the mutations across the genome and the genetic basis underlying egg production traits in egg-laying ducks.

## Results and discussion

### Phenotype statistics and genome variants

Statistics results of egg number (EN) and egg weight (EW) traits between HEN and LEN duck number ducks at 71 weeks are shown in Table S1. Egg number and total egg weight in the HEN group were significantly increased to 22.97% (*P* = 0.007) and 27.38% (*P* = 0.011), respectively, compared with the LEN group. There was no significant difference in EW between the two groups.

A total of 82.69 Gb raw base reads were obtained from three HEN and LEN ducks, shown in Table S2. Clean data reads of each sample ranging from 12.42 to 15.13 Gb were acquired after the quality control. The sequencing quality was high with Q20 and Q30 ratio greater than 92%. Mapping rate, average genome depth and coverage were obtained through clean reads mapped to the duck reference genome in Table S3, with the mapping rate of each sample more than 94%. The average sequencing depth ranged from 9.02 to 10.76 times and four times coverage ranged from 91.80% to 94.89%. The sequencing quality, mapping rate and average sequencing depth met the requirements for detecting the genomic variations.

A total of 7,243,250 SNPs and 864,777 indels were identified in the Shan-ma duck genome in Table 1. The number of SNPs and indels in the Shan-ma duck detected in this study were less than other duck types^15,16^, because the trial birds were full sibling individuals of one breed. In this study, the number of SNPs in intergenic, intronic and exonic regions including exonic stop gain, exonic stop loss, exonic synonymous, and exonic non-synonymous were 3,531,432 (52.21%), 3,232,152 (47.79%) and 114,824 (1.70%), respectively.

**Table 1:** Statistical results of SNP and indel detection and annotation.

SNPs		Indels	
Category	Number of SNPs	Category	Number of indels
Upstream	95,612	Upstream	11,787
Exonic stop gain	421	Exonic Stop gain	59
Exonic stop loss	49	Exonic Stop loss	4
Exonic synonymous	79,997	Exonic Frameshift deletion	1225
Exonic non-synonymous	34,357	Exonic Frameshift insertion	910
Intronic	3,232,152	Exonic Non-frameshift deletion	598
Splicing	358	Exonic Non-frameshift insertion	361
UTR3	112,171	Intronic	397,769
UTR5	45,479	Splicing	197
UTR5; UTR3	34	Downstream	13,722
Downstream	98,061	Upstream/Downstream	659
upstream/downstream	4619	Intergenic	417,356
Intergenic	3,531,432	Insertion	365,133
ts	5,131,106	Deletion	499,644
tv	2,112,144	Total	864,777
ts/tv	2429		
Total	7,243,250		

In the exonic region, 79,997 (69.67%) of the SNPs were synonymous mutations, 34,357 (29.92%) were non-synonymous mutations, 421 (0.37%) were stop gain and 49 (0.04%) were stop loss. The transition to transversion ratio (Ts/Tv) was 2.429, which indicated the SNP quality was comparable to human and animal Ts/Tv ranges of 2.0 to 2.4 (Li et al., 2020). For the indels, 365,133 (42.22%) were insertion and 499,644 (57.78%) were deletion variants. In the exonic region, indels consisted of 2135 frameshift, 959 non-frameshift, 54 stop gain and nine stop-loss variants.

### Common differential variations, annotation, and function enrichment analyses

A total of 65,535 common differential SNPs and 4,702 common differential indels were identified between the HEN and LEN groups. The detailed annotated information is shown in Tables S4 and S5. The common differential SNPs and indels participated in 2027 and 954 genes, respectively, including 2417 common genes. These 2417 genes with enriched gene ontology (GO) terms and Kyoto encyclopedia of genes and genome (KEGG) pathways were determined through annotation using the online analysis tool Database for Annotation, Visualization and Integrated Discovery (DAVID, version v2021q4) (https://david.ncifcrf.gov/)^17^. Fourteen significantly enriched GO terms with FDR corrected *P* value < 0.05 were identified, including two molecular function GO terms, including nucleic acid binding transcription factor activity and transcription factor activity sequence-specific DNA binding, two cell components GO terms, including plasma membrane and cell periphery and ten biological process GO terms, including homophilic cell adhesion via plasma membrane adhesion molecules, signaling, cell communication, regulation of signaling, single organism signaling, regulation of cell communication, regulation of signal transduction, signal transduction, cellular response to organic substance and cell–cell adhesion via plasma-membrane adhesion molecules in Table S6.

Fourteen KEGG pathways were significantly enriched (FDR corrected *P* value < 0.05) as shown in Table S7. Four of 14 pathways, including MAPK signaling, Wnt signaling, melanogenesis and calcium signaling pathways were associated with poultry egg production in previous studies^18–20^. Because nine SNPs including exon 3: c.C855T:p.L285L, c.T852C:p.I284I, c.C741T:p.N247N, c.G645C:p.P215P, c.A435G:p.R145R, c.A405G:p.T135T, c.T372C:p.N124N, c.A329G:p.N110S and exon 4: c.A1437G:p.K479K located at two exons of *FZD6* were found using whole-genome resequencing and enriched in both Wnt signaling and melanogenesis signaling pathways, it was selected for further study.

### Association study

To further confirm the association between *FZD6* and egg production traits in the Shan-ma duck, *FZD6* in 329 female ducks was genotyped by Sequenom MassARRAY technology. The primers of *FZD6* genotyped used in Sequenom MassARRAY platform are shown in Table S8. Fifty-three SNPs were found and 35 retained for further association study after sixteen SNPs were removed because the HWE *P* value was less than or equal to 4.86 × 10^–5^ and two SNPs excluded because MAF was less than 0.05 in Table 2. A total of 64 associations were identified between 29 SNPs of *FZD6* and four traits, EP17, EP18, EP19 and EW59 in Table 3. Twenty-one SNPs associated with EP17 were identified, with the minor allele of 14 SNPs including A1546176G, A1546196T, C1546217T, A1546400G, G1546616A, A1547351G, A1547887T, C1548016T, C1548761T, G1564507A, C1567012T, A1567491G, T1570377C, and G1571845C which increased EP17 and the minor allele of seven SNPs T1551159C, G1553772A, A1553775G, C1553982G, T1554192C, T1554298C and C1558458T decreased EP17. Ten SNPs associated with EP18 were found, including the minor allele of four SNPs A1546176G, A1546196T, C1546217T and C1548016T, which increased EP18 and the minor allele of six SNPs T1551159C, C1553982G, T1554298C, C1558458T, A1562579G and A1571291G, which decreased EP18. Seventeen SNPs associated with EP19 were identified, including the minor allele of four SNPs A1546176G, A1546196T, C1546217T, and C1548016T, which increased EP19 and the minor allele of 13 SNPs, including T1551159C, G1553772A, A1553775G, C1553982G, T1554192C, T1554298C, C1558458T, C1558820T, A1562579G, G1567392C, C1570687T, A1571291G and G1572493C, which decreased EP19. Sixteen SNPs associated with EW59 were identified, including the minor allele of one SNP, G1571845C, which increased EW59 and 15 SNPs, including C1558458T, T1551159C, C1553982G, A1562579G, A1571291G, G1553772A, T1554192C, A1553775G, C1570687T, C1558820T, A1570698G, T1554298C, G1558943A, G1572493C and G1567392C, which deceased EW59.

**Table 2:** Quality control filtering result of SNPs.

Name	ObsHET	PredHET	HWpval	%Geno	FamTrio	MendErr	MAF	Rating
A1546176G	0.55	0.50	0.07	99.40	0	0	0.45	
A1546196T	0.55	0.50	0.06	100.00	0	0	0.45	
C1546217T	0.55	0.50	0.07	99.10	0	0	0.45	
A1546400G	0.45	0.45	0.84	99.10	0	0	0.35	
G1546616A	0.44	0.44	1.00	99.10	0	0	0.32	
A1547351G	0.44	0.44	0.92	99.70	0	0	0.33	
A1547887T	0.44	0.44	0.97	100.00	0	0	0.33	
A1547889C	0.89	0.49	6.75 × 10^–59^	100.00	0	0	0.45	BAD
C1548016T	0.55	0.50	0.07	99.40	0	0	0.45	
C1548761T	0.44	0.44	1.00	99.40	0	0	0.32	
G1550814A	0.32	0.38	4.10 × 10^–3^	97.30	0	0	0.26	
C1551010T	0.35	0.39	0.04	99.70	0	0	0.27	
T1551159C	0.31	0.33	0.38	99.10	0	0	0.21	
G1551821A	0.51	0.47	0.12	100.00	0	0	0.37	
C1552291T	0.15	0.49	3.29 × 10^–36^	93.90	0	0	0.43	BAD
A1552824T	0.34	0.45	4.86 × 10^–59^	97.90	0	0	0.34	BAD
G1553772A	0.39	0.40	0.72	94.50	0	0	0.28	
A1553775G	0.37	0.43	0.03	100.00	0	0	0.31	
G1553886A	0.14	0.31	5.87 × 10^–20^	99.40	0	0	0.19	BAD
C1553982G	0.31	0.33	0.37	99.40	0	0	0.21	
T1554192C	0.37	0.43	0.02	99.40	0	0	0.31	
A1554255G	0.16	0.33	5.38 × 10^–17^	100.00	0	0	0.21	BAD
T1554298C	0.35	0.43	1.90 × 10^–3^	97.30	0	0	0.31	
T1557630C	0.26	0.42	2.12 × 10^–10^	99.10	0	0	0.29	BAD
T1557633C	0.01	0.01	1.00	99.40	0	0	0.00	BAD
G1557719C	0.03	0.13	2.81 × 10^–19^	98.80	0	0	0.07	BAD
C1557731G	0.07	0.08	0.03	98.20	0	0	0.04	BAD
C1557809T	0.03	0.10	5.35 × 10^–14^	98.80	0	0	0.05	BAD
A1558367G	0.33	0.37	0.03	99.40	0	0	0.25	
C1558458T	0.34	0.35	0.86	99.40	0	0	0.22	
T1558531C	0.33	0.38	0.03	100.00	0	0	0.25	
C1558820T	0.42	0.45	0.29	99.70	0	0	0.35	
G1558943A	0.42	0.46	0.20	99.70	0	0	0.35	
G1562443A	0.24	0.42	4.39 × 10^–13^	93.30	0	0	0.30	BAD
A1562579G	0.38	0.37	0.62	99.70	0	0	0.24	
G1562687C	0.21	0.36	7.01 × 10^–12^	96.40	0	0	0.24	BAD
A1562993G	0.02	0.15	4.34 × 10^–29^	99.10	0	0	0.08	BAD
G1564507A	0.42	0.44	0.50	93.90	0	0	0.33	
C1565460T	0.75	0.47	9.46 × 10^–35^	100.00	0	0	0.38	BAD
G1566061C	0.37	0.49	6.49 × 10^–6^	98.80	0	0	0.43	BAD
C1566250T	0.36	0.40	0.11	96.40	0	0	0.27	
C1567012T	0.43	0.43	1.00	100.00	0	0	0.31	
G1567392C	0.51	0.47	0.30	100.00	0	0	0.39	
A1567491G	0.40	0.44	0.16	97.60	0	0	0.32	
T1570377C	0.43	0.43	0.97	99.40	0	0	0.31	
C1570687T	0.43	0.45	0.40	100.00	0	0	0.35	
A1570698G	0.40	0.45	0.07	97.30	0	0	0.35	
A1571291G	0.34	0.35	0.70	100.00	0	0	0.22	
A1571300G	0.05	0.34	8.68 × 10^–48^	96.40	0	0	0.22	BAD
G1571845C	0.46	0.50	0.17	99.40	0	0	0.45	
A1572228C	0.03	0.12	3.43 × 10^–17^	98.50	0	0	0.06	BAD
A1572241G	0.21	0.50	1.94 × 10^–25^	97.00	0	0	0.46	BAD
G1572493C	0.51	0.47	0.12	99.40	0	0	0.37	

*ObsHET* the SNP's observed heterozygosity, *PredHET* the SNP's predicted heterozygosity, *HWpval* Hardy–Weinberg equilibrium *P* value, *%Geno* the percentage of non-missing genotypes for the SNPs, *FamTrio* the number of fully genotyped family trios for the SNPs, *MendErr* the number of observed Mendelian inheritance error, *MAF* minimum allele frequency.

**Table 3:** Results of associations of the *FZD6* gene with egg production traits.

Trait	SNP	BP	A1	TEST	NMISS	BETA	STAT	*P* value
EP17	A1547351G	1,547,351	G	ADD	328	0.52	2.98	2.77 × 10^–2^
EP17	C1553982G	1,553,982	G	ADD	327	− 0.57	− 2.84	2.77 × 10^–2^
EP17	A1547887T	1,547,887	T	ADD	329	0.50	2.84	2.77 × 10^–2^
EP17	G1546616A	1,546,616	A	ADD	326	0.50	2.80	2.77 × 10^–2^
EP17	C1548761T	1,548,761	T	ADD	327	0.49	2.77	2.77 × 10^–2^
EP17	T1551159C	1,551,159	C	ADD	326	− 0.55	− 2.76	2.77 × 10^–2^
EP17	T1554298C	1,554,298	C	ADD	320	− 0.44	− 2.70	2.77 × 10^–2^
EP17	A1546400G	1,546,400	G	ADD	326	0.47	2.68	2.77 × 10^–2^
EP17	C1546217T	1,546,217	T	ADD	326	0.48	2.66	2.77 × 10^–2^
EP17	A1546196T	1,546,196	T	ADD	329	0.48	2.63	2.77 × 10^–2^
EP17	A1546176G	1,546,176	G	ADD	327	0.47	2.61	2.77 × 10^–2^
EP17	C1548016T	1,548,016	T	ADD	327	0.47	2.61	2.77 × 10^–2^
EP17	A1553775G	1,553,775	A	ADD	329	− 0.42	− 2.49	3.54 × 10^–2^
EP17	T1554192C	1,554,192	C	ADD	327	− 0.41	− 2.44	3.85 × 10^–2^
EP17	T1570377C	1,570,377	C	ADD	327	0.43	2.40	3.98 × 10^–2^
EP17	C1567012T	1,567,012	T	ADD	329	0.42	2.37	3.98 × 10^–2^
EP17	G1564507A	1,564,507	A	ADD	309	0.42	2.35	3.98 × 10^–2^
EP17	A1567491G	1,567,491	G	ADD	321	0.39	2.28	4.42 × 10^–2^
EP17	C1558458T	1,558,458	T	ADD	327	− 0.44	− 2.26	4.42 × 10^–2^
EP17	G1553772A	1,553,772	G	ADD	311	− 0.42	− 2.25	4.42 × 10^–2^
EP17	G1571845C	1,571,845	C	ADD	327	0.35	2.21	4.66 × 10^–2^
EP18	C1553982G	1,553,982	G	ADD	327	− 1.12	− 3.36	1.95 × 10^–2^
EP18	T1551159C	1,551,159	C	ADD	326	− 1.10	− 3.29	1.95 × 10^–2^
EP18	C1558458T	1,558,458	T	ADD	327	− 0.94	− 2.87	3.60 × 10^–2^
EP18	C1546217T	1,546,217	T	ADD	326	0.83	2.73	3.60 × 10^–2^
EP18	T1554298C	1,554,298	C	ADD	320	− 0.75	− 2.70	3.60 × 10^–2^
EP18	A1546196T	1,546,196	T	ADD	329	0.82	2.68	3.60 × 10^–2^
EP18	A1546176G	1,546,176	G	ADD	327	0.81	2.66	3.60 × 10^–2^
EP18	C1548016T	1,548,016	T	ADD	327	0.81	2.66	3.60 × 10^–2^
EP18	A1562579G	1,562,579	G	ADD	328	− 0.84	− 2.56	4.22 × 10^–2^
EP18	A1571291G	1,571,291	G	ADD	329	− 0.81	− 2.49	4.64 × 10^–2^
EP19	C1553982G	1,553,982	G	ADD	327	− 1.68	− 3.83	3.54 × 10^–3^
EP19	T1551159C	1,551,159	C	ADD	326	− 1.65	− 3.76	3.54 × 10^–3^
EP19	C1558458T	1,558,458	T	ADD	327	− 1.40	− 3.25	1.50 × 10^–2^
EP19	T1554298C	1,554,298	C	ADD	320	− 1.14	− 3.12	1.73 × 10^–2^
EP19	A1562579G	1,562,579	G	ADD	328	− 1.29	− 2.99	2.09 × 10^–2^
EP19	A1571291G	1,571,291	G	ADD	329	− 1.23	− 2.88	2.48 × 10^–2^
EP19	A1553775G	1,553,775	A	ADD	329	− 1.00	− 2.73	3.34 × 10^–2^
EP19	T1554192C	1,554,192	C	ADD	327	− 0.99	− 2.68	3.44 × 10^–2^
EP19	C1546217T	1,546,217	T	ADD	326	1.01	2.51	3.98 × 10^–2^
EP19	G1572493C	1,572,493	C	ADD	327	− 1.00	− 2.50	3.98 × 10^–2^
EP19	A1546196T	1,546,196	T	ADD	329	0.98	2.45	3.98 × 10^–2^
EP19	A1546176G	1,546,176	G	ADD	327	0.98	2.43	3.98 × 10^–2^
EP19	C1548016T	1,548,016	T	ADD	327	0.98	2.43	3.98 × 10^–2^
EP19	G1553772A	1,553,772	G	ADD	311	− 0.99	− 2.42	3.98 × 10^–2^
EP19	G1567392C	1,567,392	C	ADD	329	− 0.91	− 2.35	4.49 × 10^–2^
EP19	C1558820T	1,558,820	T	ADD	328	− 0.85	− 2.29	4.70 × 10^–2^
EP19	C1570687T	1,570,687	C	ADD	329	− 0.85	− 2.29	4.70 × 10^–2^
EW59	C1558458T	1,558,458	T	ADD	327	− 102.40	− 4.67	9.21 × 10^–5^
EW59	C1553982G	1,553,982	G	ADD	327	− 102.20	− 4.56	9.21 × 10^–5^
EW59	T1551159C	1,551,159	C	ADD	326	− 102.20	− 4.54	9.21 × 10^–5^
EW59	A1562579G	1,562,579	G	ADD	328	− 93.75	− 4.30	2.02 × 10^–4^
EW59	A1571291G	1,571,291	G	ADD	329	− 89.86	− 4.13	3.26 × 10^–4^
EW59	A1553775G	1,553,775	A	ADD	329	− 73.10	− 3.91	5.41 × 10^–4^
EW59	T1554192C	1,554,192	C	ADD	327	− 73.30	− 3.90	5.41 × 10^–4^
EW59	C1570687T	1,570,687	C	ADD	329	− 72.77	− 3.87	5.41 × 10^–4^
EW59	T1554298C	1,554,298	C	ADD	320	− 70.05	− 3.83	5.41 × 10^–4^
EW59	C1558820T	1,558,820	T	ADD	328	− 71.79	− 3.83	5.41 × 10^–4^
EW59	A1570698G	1,570,698	A	ADD	320	− 70.92	− 3.76	6.06 × 10^–4^
EW59	G1553772A	1,553,772	G	ADD	311	− 77.48	− 3.75	6.06 × 10^–4^
EW59	G1558943A	1,558,943	A	ADD	328	− 69.39	− 3.73	6.06 × 10^–4^
EW59	G1572493C	1,572,493	C	ADD	327	− 59.82	− 2.95	8.65 × 10^–3^
EW59	G1571845C	1,571,845	C	ADD	327	47.65	2.65	1.98 × 10^–2^
EW59	G1567392C	1,567,392	C	ADD	329	− 51.25	− 2.61	2.09 × 10^–2^

A1, tested allele (minor allele); ADD, additive effects of SNPs, for the additive effects of SNPs, the direction of the regression coefficient represents the effect of each extra minor allele (a positive regression coefficient means that the minor allele increases traits mean).

*BETA* regression coefficient, *STAT* coefficient t-statistic, *N* number of non-missing individuals included in analysis, *P value* asymptotic *P* value for t-statistic after FDR correction.

The gene *FZD6* is a member of the frizzled gene family, which encodes frizzled class receptor 6, which is a WNT signaling protein receptor. The WNT-frizzled selectivity plays a significant role in developmental biology, stem cell regulation oncogenesis and human disease^21^. In human disease, *FZD6* is a core gene that is strongly linked to human ovarian cancer^22^. In animal reproduction, secreted frizzled-related protein completes the preparation for the next reproduction process during the transition from the young to the egg-laying phase^23^ and WNT-FZD6 interaction regulates the follicle-stimulating hormone selection for dominant follicle^24^. Based on above-mentioned information, it was believed that *FZD6* could be considered a promising candidate gene for the egg production traits, which needs to be validated in large duck populations.

In Summary, whole genome variants, including 7,243,250 SNPs and 864,777 indels from between high- and low-egg number ducks were identified using high-throughput sequencing technology, with 65,535 common differential SNPs and 4,702 common differential indels also identified by comparing sequences from the two groups of ducks, which participated in 2,027 and 954 genes, respectively, including 2,417 common genes. These genes were significantly enriched 14 GO terms and 14 KEGG pathways. Four of 14 signaling pathways, including the MAPK, Wnt, melanogenesis and calcium signaling pathways have been shown to be related to poultry egg production in previous studies. An association of *FZD6* with egg production traits has been verified as 29 SNPs were associated with four traits, EP17, EP18, EP19, and EW59. Importantly, pathways and candidate genes identified in this study will not only provide a new insight into the genetic basis underlying egg production traits in ducks but also improve better understanding the genetic architecture and molecular mechanisms of these traits in poultry.

## Methods

### Birds and samples

High-yield line Longyan Shan-ma ducks were raised in cages under the same environmental and nutritional conditions at the Longyan Shan-ma Duck Original Breeding Farm. Three high-egg (HEN) and three low-egg producing (LEN) female ducks from one full-sib family were used in this study. Blood was sampled from the brachial vein using citrate-based anticoagulant syringes before the ducks were euthanized by electrical stunning and exsanguination. The blood samples were snap-frozen in liquid nitrogen then held at − 80 °C until used.

### Whole-genome resequencing and quality control

The genomic DNA (gDNA) of blood samples was extracted using the phenol–chloroform method. The integrity of the DNA was estimated using electrophoresis in a 1% agarose gel and the purity of DNA was assessed using a NanoDrop 2000 spectrophotometer (Thermo Fisher, Foster City, CA, USA). The concentration of DNA was measured using an Invitrogen Qubit 2.0 fluorimeter (Thermo Fisher Scientific, Foster City, CA, USA). The OD260/OD280 ratios were between 1.8 and 2.0 and the concentration above 1.5 μg of each DNA sample were used to construct the DNA libraries. The DNA samples were randomly interrupted using a Covaris crusher (S220; Covaris, LLC, Woburn, MA, USA) to create fragments of 350 bp in length. The DNA Libraries were constructed using a TruSeq Library Construction Kit (Illumina Inc., San Diego, CA, USA) following the manufacturer's instructions. The DNA library was sequenced on the HiSeq 2500 high-throughput sequencing platform (Illumina Inc., San Diego, CA, USA).

Raw data reads were obtained by sequencing and clean data reads were acquired following the quality control (QC) procedure to remove unusable reads. Usable reads contained the Illumina library construction adapters, more than 10% unknown N bases and one end of the read had to contain more than 50% of low-quality bases, with a sequencing quality value less than or equal to five.

### Detection and annotation of genomic variants

All clean reads were aligned to the reference duck genome (BGI_duck_1.0) using Burrows–Wheeler aligner (BWA) software (version 0.7.8-r455) with default parameters^25^. The SNPs and indels were detected using the SAMtools (version 0.1.19)^26^ with the parameters as “-q 1 -C 50 -m 2 -F 0.002 -d 1000” and the filtering criteria “the mapping quality > 20 and the depth of the variate position > 4”. The functional annotation of these variants was carried out using ANNOVAR (version 2013Aug23)^27^ and known genes and region annotations were determined using the UCSC genome browser database^28^.

### Differential variants, annotation, and function enrichment analyses

To identify differential variants across genomes between high- and low-egg producing ducks, the SNPs with the same genotype were distinguished in each group first, then differential variants and then the above-mentioned differential or “common differential variants”^29^ were determined between the two groups. Genes located within 500 kb with these common differential variants were annotated.

The GO enrichment analyses for the genes of the common differential variants were conducted using the DAVID v6.8 online server (https://david.ncifcrf.gov/home.jsp)^17^. The KEGG pathway function enrichment analyses for the genes of the common differential variants were performed in web-based software Kobas 3.0 (http://kobas.cbi.pku.edu.cn/)^30,31^. The copyright permission (No. 221304) to publish the corresponding KEGG pathways was officially granted by Kanehisa Laboratories^32–34^. False discovery rates (FDR) with a corrected *P* value < 0.05 were considered significant for GO terms and pathways.

### Association study of *FZD6* with egg number traits

Based on the reproductive physiological function of genes in the pathways, *FZD6* was used for the further association study with 73 egg production traits in 329 female ducks, as seen in Table 4. The region 1,546,176 to 1,572,493 bp on chromosome 2 of *FZD6* was genotyped by the Sequenom MassARRAY platform (Sequenom, San Diego, CA, USA), according to the manufacturer’s instructions. Quality control of SNP genotyping data were estimated by Haploview 4.1 software^35^ and any with a minimum allele frequency (MAF) of less than 0.05 and a Hardy–Weinberg equilibrium (HWE) test *P* value < 1.0 × 10^–4^ were excluded. Associations between SNPs and egg production traits were analyzed with the general linear model (GLM) in PLINK v1.90^36^ using the following model:

**Table 4:** The descriptive statistics of body weight, egg production and egg weight traits.

Trait	N	Mean	SD	Min	Max	CV
BW0 (g)	329	38.43	1.09	34.80	42.10	2.84
BW17 (kg)	328	1.34	0.06	1.12	1.51	4.61
BW43 (kg)	326	1.45	0.08	1.24	1.70	5.77
BW72 (kg)	325	1.46	0.13	1.13	1.86	8.92
EW20	314	53.95	5.87	32.40	75.40	10.89
EW26	316	62.82	4.42	51.43	76.88	7.03
EW32	312	67.33	4.46	53.51	80.26	6.62
EW38	325	70.16	4.50	56.73	85.55	6.41
EW41	320	71.67	4.61	55.70	84.87	6.43
EW44	326	71.53	4.26	60.30	82.38	5.96
EW50	315	71.80	5.08	54.65	88.25	7.07
EW56	306	72.21	5.00	57.10	85.44	6.92
EW59	312	70.82	4.73	55.80	84.50	6.68
EW65	316	71.04	4.83	55.53	85.50	6.80
EW68	286	69.36	5.01	54.00	84.45	7.22
EW71	273	69.57	5.08	56.46	87.60	7.30
EWA	329	68.49	3.88	57.50	82.22	5.67
EW	327	22.87	2.69	13.70	30.42	11.76
EP17	329	4.72	2.11	0.00	8.00	44.80
EP18	329	10.09	3.54	0.00	15.00	35.12
EP19	329	15.66	4.67	0.00	22.00	29.83
EP20	327	21.69	5.39	2.00	29.00	24.82
EP21	326	27.83	6.03	6.00	36.00	21.67
EP22	328	33.80	7.24	7.00	43.00	21.41
EP23	326	40.29	7.62	12.00	50.00	18.90
EP24	324	46.67	7.95	19.00	57.00	17.04
EP25	325	52.18	9.02	19.00	64.00	17.29
EP26	322	58.64	9.20	27.00	71.00	15.69
EP27	323	65.03	9.78	30.00	78.00	15.04
EP28	324	71.42	10.34	33.00	85.00	14.48
EP29	324	78.02	10.67	40.00	92.00	13.68
EP30	324	84.55	11.11	47.00	99.00	13.14
EP31	326	90.80	12.15	46.00	106.00	13.38
EP32	325	97.27	12.61	52.00	113.00	12.97
EP33	325	103.47	13.32	58.00	120.00	12.87
EP34	325	109.84	13.97	58.00	127.00	12.72
EP35	323	116.62	13.91	69.00	134.00	11.93
EP36	324	122.88	14.66	69.00	141.00	11.93
EP37	324	129.41	15.03	75.00	148.00	11.61
EP38	325	135.77	15.70	78.00	155.00	11.57
EP39	325	142.41	15.89	84.00	162.00	11.16
EP40	324	149.22	15.72	92.00	169.00	10.53
EP41	324	155.73	15.85	99.00	176.00	10.18
EP42	324	162.15	16.27	103.00	182.00	10.04
EP43	324	168.58	16.78	109.00	189.00	9.95
EP44	323	175.27	16.85	113.00	196.00	9.61
EP45	323	181.91	17.09	120.00	203.00	9.40
EP46	323	188.59	17.23	125.00	210.00	9.14
EP47	323	195.24	17.39	132.00	217.00	8.91
EP48	321	202.22	16.99	144.00	224.00	8.40
EP49	321	208.69	17.25	151.00	231.00	8.27
EP50	321	215.13	17.56	156.00	238.00	8.16
EP51	321	221.55	17.82	161.00	245.00	8.04
EP52	321	227.72	18.07	164.00	252.00	7.94
EP53	321	233.87	18.43	169.00	259.00	7.88
EP54	322	239.67	19.49	169.00	266.00	8.13
EP55	322	245.70	20.12	175.00	273.00	8.19
EP56	321	252.07	20.40	182.00	280.00	8.09
EP57	321	258.15	21.07	188.00	287.00	8.16
EP58	321	264.25	21.75	191.00	294.00	8.23
EP59	321	270.17	22.36	195.00	301.00	8.28
EP60	321	275.87	22.99	198.00	308.00	8.33
EP61	321	281.79	23.60	202.00	315.00	8.38
EP62	321	287.75	24.27	209.00	319.00	8.44
EP63	321	293.69	25.03	213.00	326.00	8.52
EP64	321	299.60	25.73	214.00	332.00	8.59
EP65	321	305.24	26.52	216.00	339.00	8.69
EP66	322	310.49	27.91	216.00	346.00	8.99
EP67	322	315.88	28.78	217.00	353.00	9.11
EP68	322	321.16	29.72	218.00	360.00	9.25
EP69	322	326.10	30.44	220.00	367.00	9.33
EP70	322	330.91	31.25	222.00	373.00	9.44
EP71	322	335.55	32.15	224.00	380.00	9.58
EP21,22	327	12.18	3.05	1.00	15.00	25.04
EP43,44	325	12.86	2.55	0.00	14.00	19.82
EP56,57	322	12.19	3.30	0.00	14.00	27.07
EP70,71	323	9.42	4.66	0.00	14.00	49.51

BW0, BW17, BW43 and BW72, body weight at birth date, 17, 43 and 72 weeks; EW20, EW26, EW32, EW38, EW41, EW44, EW50, EW56, EW59, EW65 and EW68, egg weight at 20, 26, 32, 38, 41, 44, 50, 56, 59, 65, 68 weeks; EW71EN17 ~ EN71, egg production at 17–71 weeks; EN21,22, EN43,44, EN56,57 and EN70,71, egg production at 21–22, 43–44, 56–57, 70–71 weeks.

*SD* standard deviation, *CV* coefficient of variation.

$$Y=\mu +F+BW+G+e$$

where *Y* is the trait value, *µ* is the overall mean, *F* is the family effect, *BW* is the body weight effect, *G* is the fixed effect of genotype and *e* is the random error.

### Statistical analysis

Statistical analysis of egg production and weight traits between high- and low-egg production birds was performed with the independent-sample T test using SPSS 19.0 (IBM, Armonk, NY, USA) and the results are presented as the mean ± standard deviation (SD). For the traits used in the association study, abnormal values of these traits were excluded before analysis using Grubbs’ method and the descriptive statistics were analyzed by Minitab 17.0 Statistical Software (Minitab Inc., State College, PA, USA).

### Ethics statement

This study protocol was approved by the Longyan University Ethics Committee. All animal studies were conducted in accordance with the Guidelines for Experimental Animals established by the Ministry of Science and Technology (Beijing, China). This study is also reported in accordance with the ARRIVE guidelines (https://arriveguidelines.org).

## Supplementary Information

Supplementary Information.

## Data Availability

All the data is available on reasonable request from the corresponding author.

## References

[1] Wang C (2012). Molecular cloning, expression profile, polymorphism and the genetic effects of the dopamine D1 receptor gene on duck reproductive traits. Mol. Biol. Rep..

[2] Yuan J (2015). Identification of promising mutants associated with egg production traits revealed by genome-wide association study. PLoS ONE.

[3] Sitzenstock F (2013). Efficiency of genomic selection in an established commercial layer breeding program. Genet. Sel. Evol..

[4] Goto T, Tsudzuki M (2017). Genetic mapping of quantitative trait loci for egg production and egg quality traits in chickens: A review. J. Poult. Sci..

[5] Hu ZL, Park CA, Reecy JM (2019). Building a livestock genetic and genomic information knowledgebase through integrative developments of Animal QTLdb and CorrDB. Nucleic Acids Res..

[6] Liu Z (2019). Genome-wide association analysis of egg production performance in chickens across the whole laying period. BMC Genet..

[7] Lin RL, Chen HP, Rouvier R, Marie-Etancelin C (2016). Genetic parameters of body weight, egg production, and shell quality traits in the Shan Ma laying duck (Anas platyrhynchos). Poult Sci..

[8] Xu J (2017). Molecular characterization, expression profile of the FSHRgene and its association with egg production traits in muscovy duck. J. Genet..

[9] Feng P (2018). Polymorphisms of melatonin receptor genes and their associations with egg production traits in Shaoxing duck. Asian-Australas J. Anim. Sci..

[10] Purwantini D (2020). Prolactin gene polymorphisms and associations with reproductive traits in Indonesian local ducks. Vet. World.

[11] Li X (2020). Whole-genome sequencing identifies potential candidate genes for reproductive traits in pigs. Genomics.

[12] Qiu, M. *et al.* Whole-genome resequencing reveals aberrant autosomal SNPs affect chicken feathering rate. *Anim Biotechnol*, 1–13. 10.1080/10495398.2020.1846545 (2020). 10.1080/10495398.2020.1846545 33342337

[13] Zhou Z (2018). An intercross population study reveals genes associated with body size and plumage color in ducks. Nat. Commun..

[14] Wen J (2021). Genomic scan revealed KIT gene underlying white/gray plumage color in Chinese domestic geese. Anim. Genet.

[15] Gu L (2020). Genetic characteristics of Jiaji Duck by whole genome re-sequencing. PLoS ONE.

[16] Lee D (2020). Population analysis of the Korean native duck using whole-genome sequencing data. BMC Genomics.

[17] Huang DW (2007). DAVID Bioinformatics Resources: Expanded annotation database and novel algorithms to better extract biology from large gene lists. Nucleic Acids Res..

[18] Folaniyi, B. S. *et al.* Hypothalamic and ovarian transcriptome profiling reveals potential candidate genes in low and high egg production of White Muscovy Ducks (Cairina Moschata). *Poultry Sci.* 101310 (2021). 10.1016/j.psj.2021.101310 PMC8322464 34298381

[19] Zhang T (2019). Transcriptome analysis of ovary in relatively greater and lesser egg producing Jinghai Yellow Chicken. Anim. Reprod. Sci..

[20] Tao Z (2017). Comparative transcriptomic analysis of high and low egg-producing duck ovaries. Poult. Sci..

[21] Kilander MB, Dahlstrom J, Schulte G (2014). Assessment of Frizzled 6 membrane mobility by FRAP supports G protein coupling and reveals WNT-Frizzled selectivity. Cell Signal.

[22] Kumar SU, Kumar DT, Siva R, Doss CGP, Zayed H (2019). Integrative bioinformatics approaches to map potential novel genes and pathways involved in ovarian cancer. Front. Bioeng. Biotechnol..

[23] Ren J (2017). Divergently expressed gene identification and interaction prediction of long noncoding RNA and mRNA involved in duck reproduction. Anim. Reprod. Sci..

[24] Gupta PS (2014). Regulation and regulatory role of WNT signaling in potentiating FSH action during bovine dominant follicle selection. PLoS ONE.

[25] Li H, Durbin R (2009). Fast and accurate short read alignment with Burrows-Wheeler transform. Bioinformatics.

[26] Li H (2009). The sequence alignment/map format and SAMtools. Bioinformatics.

[27] Wang K, Li M, Hakonarson H (2010). ANNOVAR: functional annotation of genetic variants from high-throughput sequencing data. Nucleic Acids Res.

[28] Navarro Gonzalez, J. *et al.* The UCSC Genome Browser database: 2021 update. *Nucleic Acids Res* **49**, D1046–D1057. 10.1093/nar/gkaa1070 (2021). 10.1093/nar/gkaa1070 PMC7779060 33221922

[29] Jiang J (2016). Whole-genome resequencing of holstein bulls for indel discovery and identification of genes associated with milk composition traits in dairy cattle. PLoS ONE.

[30] Wu J, Mao X, Cai T, Luo J, Wei L (2006). KOBAS server: A web-based platform for automated annotation and pathway identification. Nucleic Acids Res..

[31] Xie, C. *et al.* KOBAS 2.0: A web server for annotation and identification of enriched pathways and diseases. *Nucleic Acids Res.* **39**, W316–322. 10.1093/nar/gkr483 (2011). 10.1093/nar/gkr483 PMC3125809 21715386

[32] Kanehisa M, Goto S (2000). KEGG: kyoto encyclopedia of genes and genomes. Nucleic Acids Res..

[33] Kanehisa M (2019). Toward understanding the origin and evolution of cellular organisms. Protein Sci..

[34] Kanehisa M, Furumichi M, Sato Y, Ishiguro-Watanabe M, Tanabe M (2021). KEGG: Integrating viruses and cellular organisms. Nucleic Acids Res..

[35] Barrett, J. C. Haploview: Visualization and analysis of SNP genotype data. *Cold Spring Harb Protoc*. pdb ip71. 10.1101/pdb.ip71 (2009). 10.1101/pdb.ip71 20147036

[36] Purcell S (2007). PLINK: A tool set for whole-genome association and population-based linkage analyses. Am. J. Hum. Genet..

